# Design and parameter study of banana crown mechanical cutting device

**DOI:** 10.1371/journal.pone.0275365

**Published:** 2022-11-21

**Authors:** Fujun Wen, Zhengbo Zhu

**Affiliations:** 1 Guangzhou Panyu polytechnic, Guangzhou, China; 2 Jiangsu Engineering Center for Modern Agricultural Machinery and Agronomy Technology, Yanzhou University, Jiangsu, China; Sultan Qaboos University, OMAN

## Abstract

Banana mechanical crown cutting tool which is a critical component of banana crown cutting machine is designed and studied in this paper. Experiments were designed to optimize parameters of the cutting tool. Indexes are cut surface quality grade of banana crown, maximum cutting force and useful power consumption. For banana preservation, get high grade of cut surface quality is more significant than consume less energy. Results of experiments show that the optimum parameters are as follows: cutting speed is about 50–60 mm/s, number of cut sets are about 4–6, angle between thick cutter and axis of banana rachis is about 5°, width of thick cutter is about 8–14 mm, thickness of thick cutter is about 2–3 mm, edge angle of thick cutter is between 20° and 30°, width of thin cutter is about 10–14 mm and thickness of thin cutter is about 0.2–0.4mm. This study helps to make the completely mechanical postharvest treatments for banana postharvest treatments.

## 1 Introduction

According to FAO data, the global banana production was 66.0 million tons in 2000, 106.0 million tons in 2013, 106.2 million tons in 2014, and 117 million tons in 2019.Among the banana growing countries / regions in the world, India ranks first in annual output, accounting for about 26.08% of the world’s banana output; China ranks second, accounting for about 10.27% of the world’s banana production [1]. The postharvest treatments of banana mainly include harvesting, transportation, crown cutting, disinfection, cleaning, and packing [2]. All treatments are short-period for banana fresh preservation. The treatment of banana crown cutting needs to be manually operated worldwide because there is no banana crown cutting machine or mechanical tools, which result in labor-intensive and low efficiency for banana postharvest. In order to improve the productivity, banana crown cutting machine needs to be researched to make the completely mechanical postharvest treatments for banana.

Bananas are the food for people to survive, so bananas are also recognized as the fourth largest food crop in the world. At this stage, banana harvesting is dominated by manual labor, with a low degree of mechanization, especially in the banana combing link, there is no mechanical processing equipment, relying on manual processing, the mechanical chain of harvesting and processing is incomplete, and it is impossible to achieve full automation. Therefore, it is of practical significance and theoretical value to carry out research on key technologies and devices of banana mechanical combing.

The authors have studied non-destructive transportation of banana harvest [3–5], and two kinds of banana crown cutting ways which are rotary cutters way and combined cutters way have been researched based on manually operating characteristics [6–8]. Rotary cutters way means that there is two blades cut banana crown by doing rotary motion around the rachis while combined cutters way means that there are a few of blades cut banana crown at same time along the axis of rachis to separate banana crown from rachis. On account of banana bunch especially the rachis in size change, experimental results show that the rotary cutters way cannot cut banana crown well and the cut surface quality of banana crown is unqualified for banana preservation, but the way of combined cutters can get high grade of cut surface quality and can meet the requirements of mechanization for banana crown cutting. In order to optimize parameters of combined cutters, this paper take banana crown as experimental subject, and aiming at improving cut surface quality, decrease the maximum value of cutting force and reduce energy consumption.

## 2 Materials and methods

### 2.1. Mechanical structure of banana crown cutting tool by combined cutters way

**Fig 1:**
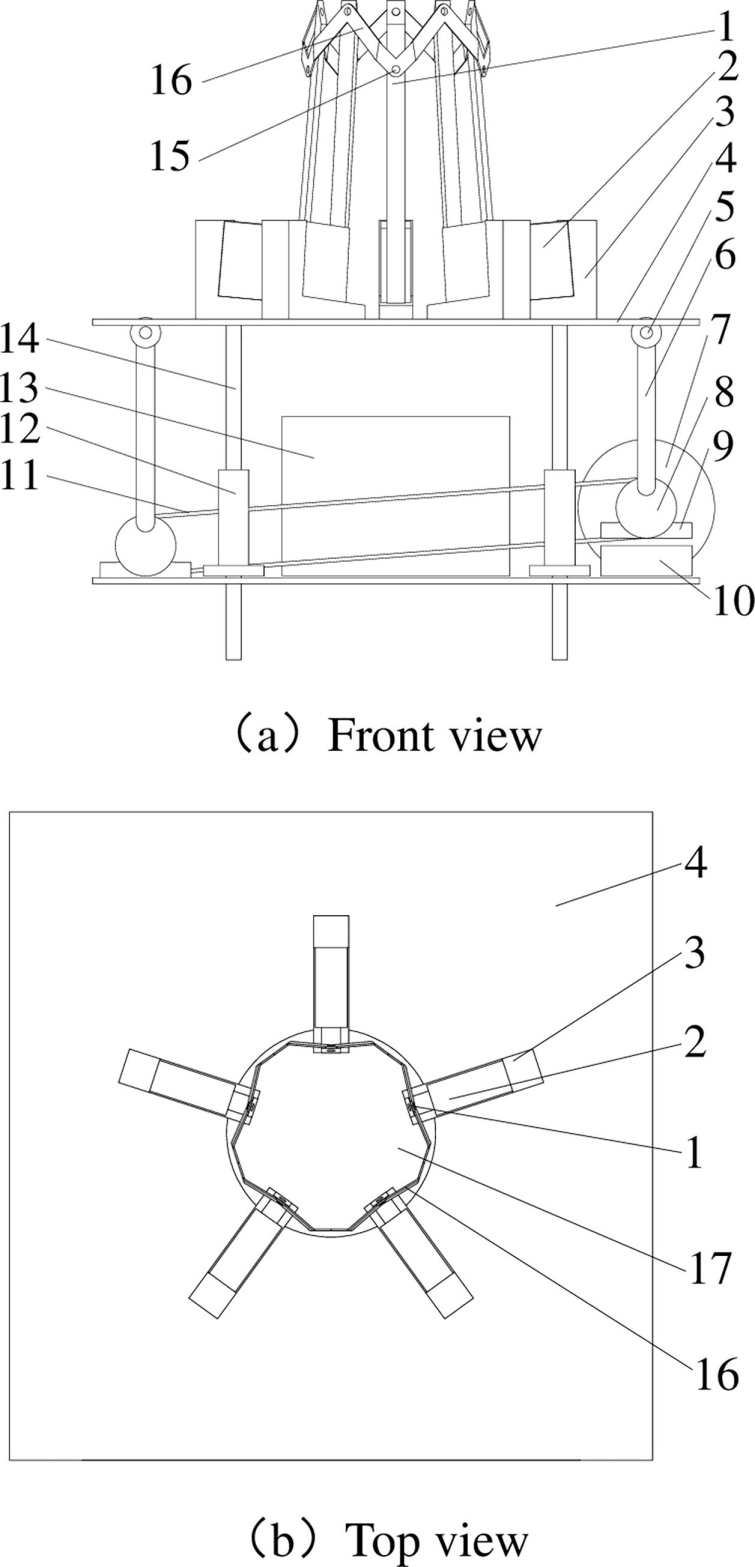
Structure diagram of banana crown cutting tool of combined cutters way. (a) Front view, (b) Top view. 1. Thick cutter, 2. Guide rod cylinder, 3. Cylinder support frame, 4. Cutter dish, 5. Pin, 6. Connecting rod, 7. Stepper motor, 8. Cam, 9. Integral bearing, 10. Block, 11. Synchronous belt, 12. Linear bearing of flange type, 13. Stepper motor controller, 14. Axis, 15. Hollow rivet, 16. Thin cutter, 17. Hole for banana rachis.

Banana mechanical combing device refers to a comprehensive banana combing machine that includes a clamping mechanism, a lifting mechanism, a combing mechanism and a transporting mechanism, prototype model of the banana crown cutting tool is designed for both efficiency and cost effectiveness.It is mainly composed of 17 devices such as rough cutter, guide rod cylinder, cylinder bracket and so on.The mechanical structure of banana crown cutting tool by combined cutters way is shown in Fig 1.

The cutters can move up and down along the axis controlled by the stepper motor controller. The combined cutters encircles the banana rachis controlled by the guide rod cylinder.

The importance parameters of the cutters consist of cutting speed, number of cut sets, angle between thick cutter and axis of banana rachis, width of thick cutter, thickness of thick cutter, edge angle of thick cutter, width of thin cutter and thickness of thin cutter.

### 2.2 Cutting force

**Fig 2:**
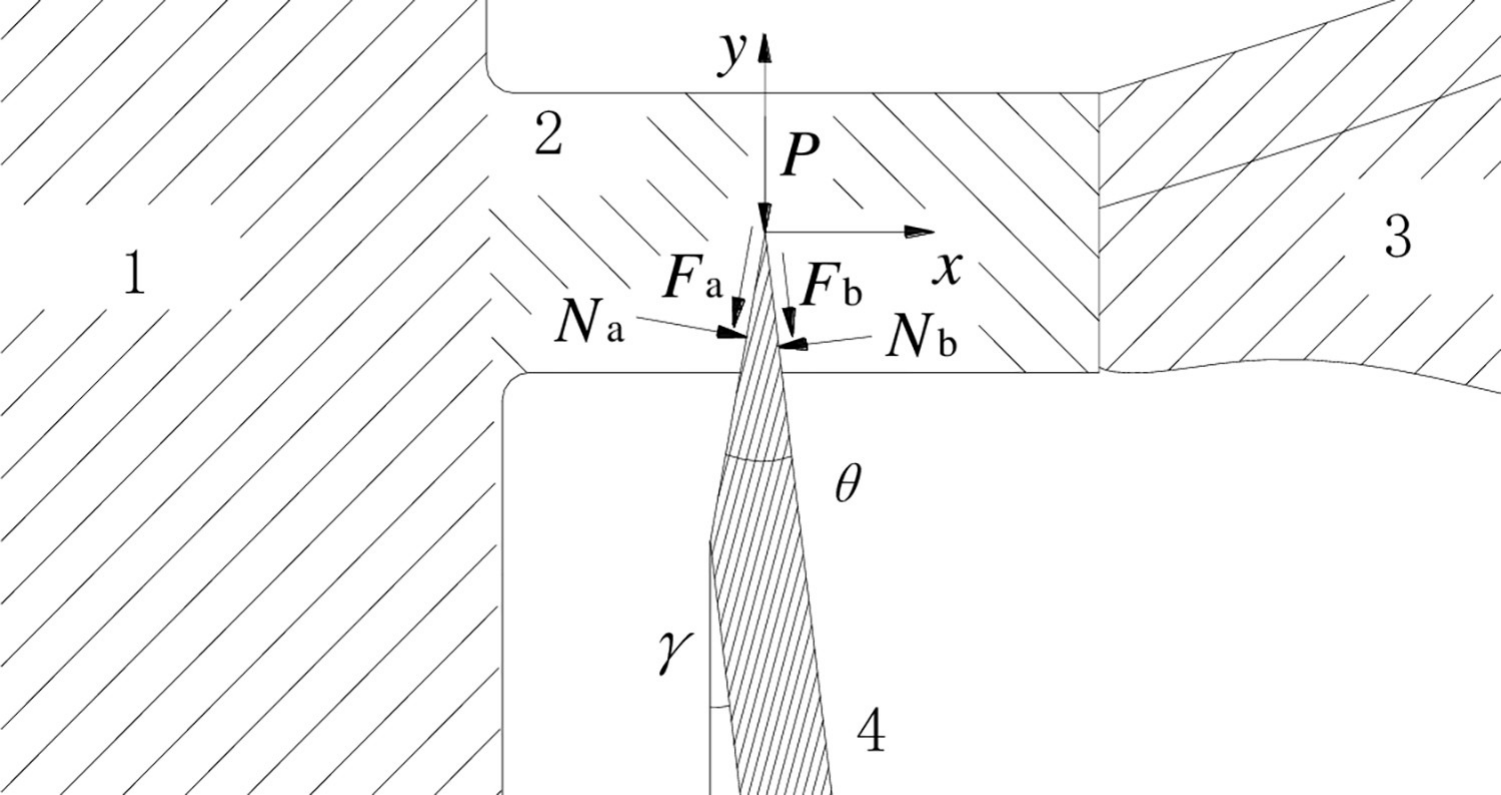
Free-body diagram of cutter cutting banana crown. 1. Banana rachis, 2. Banana crown, 3. Banana hand, 4. Cutter.

Free-body diagram of cutter cutting banana crown is shown in Fig 2.

The equations are expressed by:

$$F_{x}=N_{a}\cos(\theta -\gamma )-F_{a}sin(\theta -\gamma )+F_{b}\sin\gamma -N_{b}\cos\gamma$$ (1)

$$-F_{y}=P+F_{a}\cos(\theta -\gamma )+F_{b}\cos\gamma +N_{a}\sin(\theta -\gamma )+N_{b}\sin\gamma$$ (2)

$$F_{a}=fN_{a}$$ (3)

$$F_{b}=fN_{b}$$ (4)

where *F*_*x*_ is cutting force in *x* direction, *F*_*y*_ is cutting force in *y* direction, *P* is the cutter edge resistance, *N*_a_ and *N*_b_ are Normal loads, *F*_a_ and *F*_b_ are frictional forces, *f* is friction coefficient.

### 2.3 Materials

Forty bunches of mature green bananas (Musa AAA, ‘Baxi Jiao’) from Machong Farm, Dongguan City, Guangdong province, China, were harvested on July, from 2014 to 2021. The farm is at 23°2’24" degrees north latitude and 113°34’48" degrees east longitude. The ripening stage of banana is green [9]. Appropriate part of banana rachis between neighboring banana hand was cut to separate each banana hand. These materials were stored in cardboard boxes with polyethylene liners.

### 2.4 Methods

#### 2.4.1 Description of test device

**Fig 3:**
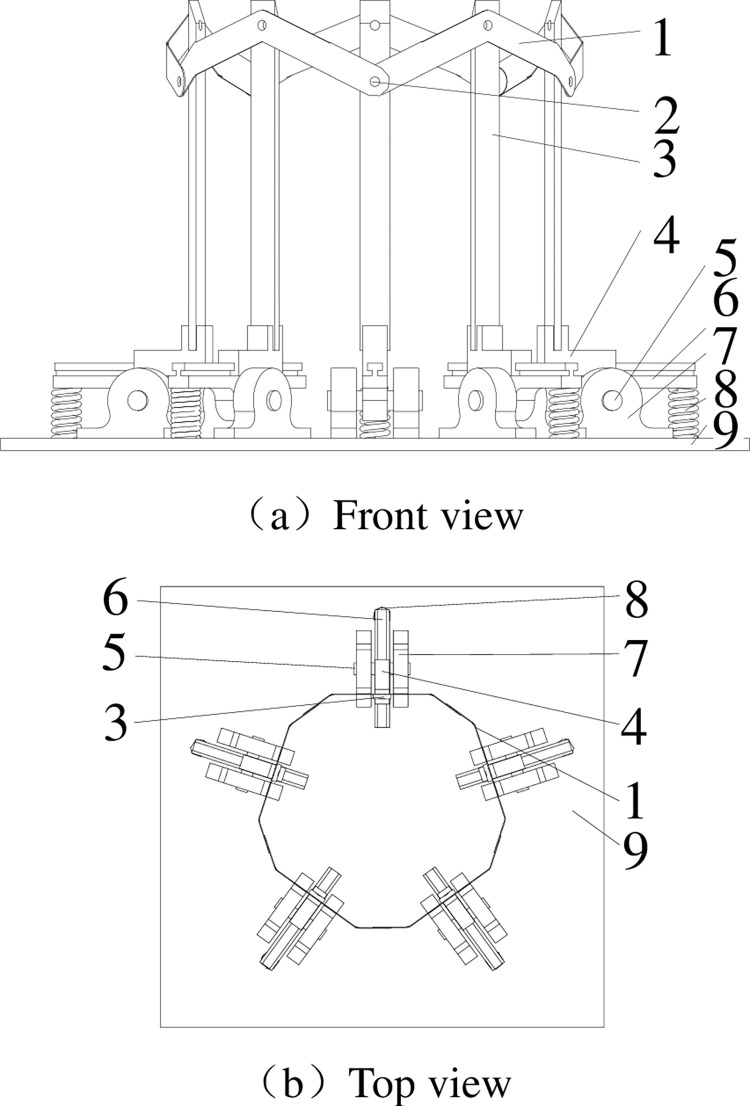
Structure diagram of test device. (a) Front view, (b) Top view. 1. Thin cutter, 2. Hollow rivets, 3. Thick cutter, 4.slider, 5. Axis, 6. sliding rail, 7. Integral bearing, 8. Screw, 9. Bottom.

An test device was designed to optimize the main parameters of the banana crown cutting tool. The main characteristics of the device are detailed in Table 1 and shown in Fig 3. It have several cut sets and a bottom. The cut sets connected in a circle and each cut set have two thin cutters, one thick cutter, one slider, one sliding rail, one spring, one axis and two pillow block bearings, and the bottom have many locating holes to fix cut set. As slider was mounted on the sliding rail and neighboring cutters were connected by hollow rivets, under the function of the spring each thick cutter could contact banana rachis despite its diameter size change.

**Table 1:** Main characteristics of test device.

Characteristics	Unit	Value
Length of bottom	mm	310
Width of bottom	mm	310
Height	mm	220
Weight	kg	3.39
Length of thick cutter	mm	190
Max diameter of banana rachis	mm	100
Min diameter of banana rachis	mm	30

#### 2.4.2 Experiment design

**Fig 4:**
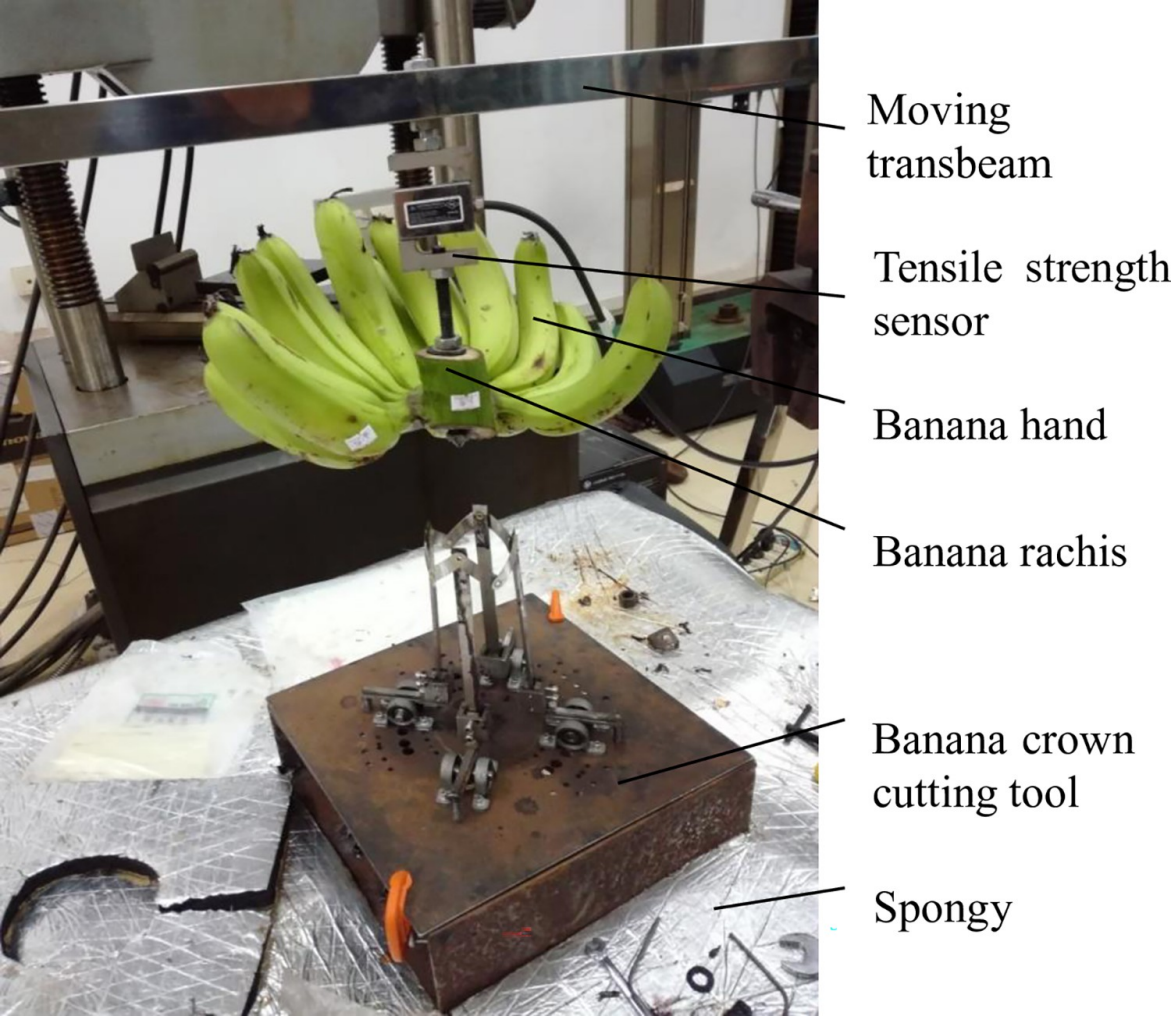
Experimental prototype.

The experiments took place in laboratory of the South China Agricultural University (Fig 4). The banana hand was fixed under the tensile strength sensor (CELTRON STC-100 kg, VISHAY CELTRON (Tianjin) TECHOLOGIES Co., LTD) which was fixed under moving transbeam. The moving transbeam can move up and down by a stepper motor (110BYG350C, Shantou Hongbaoda electrical machinery co., LTD). The tensile strength sensor can record the cutting force at 50 times per second. The prototype is mainly made by stainless steel except the thick cutter made by tool steel.

Table 2 shows the levels of factors of the device. The tests were repeated 3 times.

**Table 2:** Levels of experimental factors.

levels	cutting speed /mm·s^-1^	number of cut sets	edge angle of thick cutter /°	width of thick cutter /mm	thickness of thick cutter /mm	width of thin cutter /mm	thickness of thin cutter /mm	angle between thick cutter and axis of banana rachis /°
1	20	3	10	8	2	8	0.2	0
2	30	4	15	10	2.5	10	0.4	10
3	40	5	20	12	3	12	0.3	5
4	50	6	25	14	3.5	14		
5	60	7	30	16	4	16		

The indexes are maximum cutting force, useful power consumption and cut surface quality.

## 3 Results and discussion

### 3.1 Cutting force on displacement

**Fig 5:**
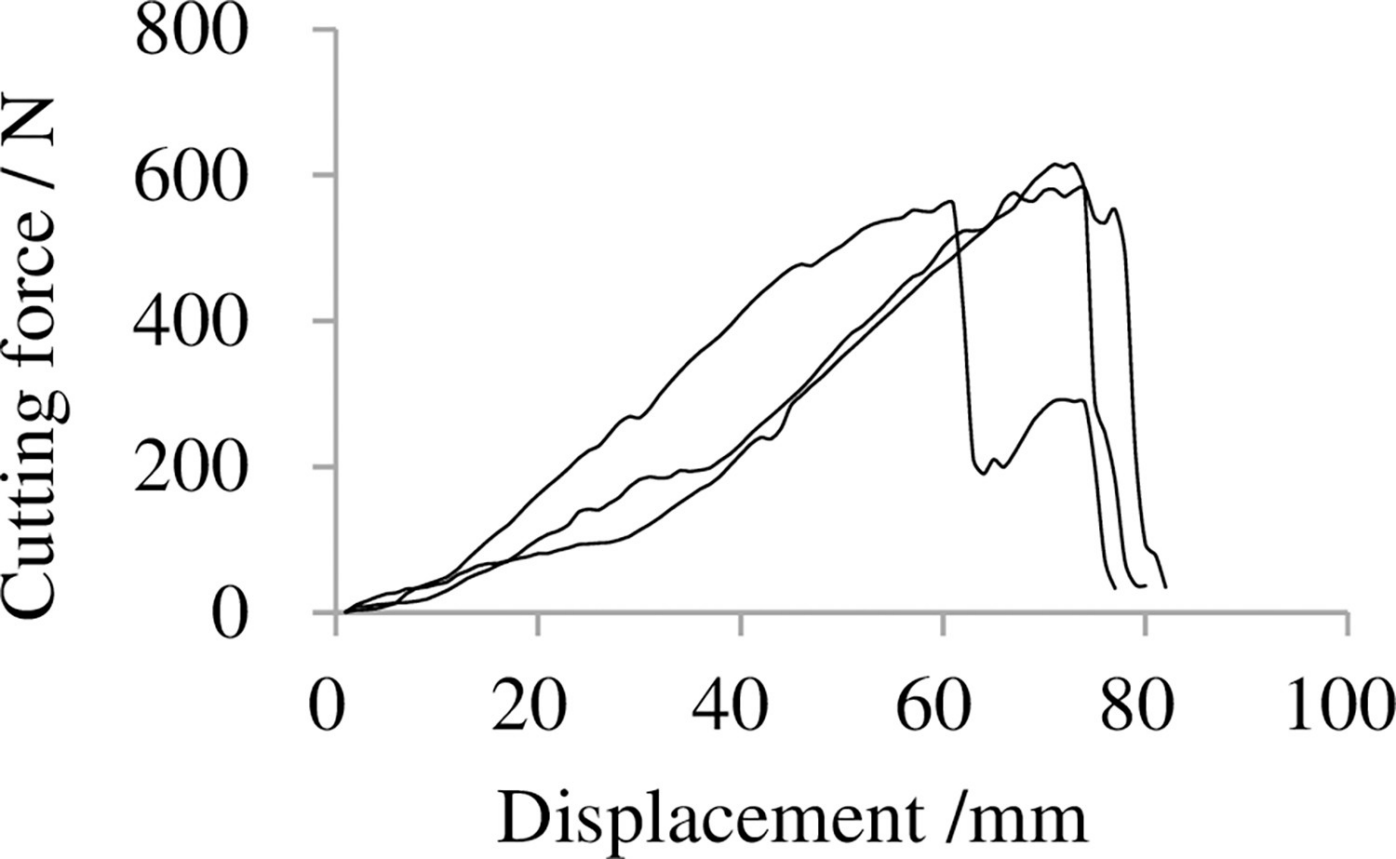
Effect of cutting force on displacement. Note: Diameter of banana rachis is between 45 and 50 mm, cutting speed is 40 mm/s, number of cut sets are 5, angle between thick cutter and axis of banana rachis is 5°, width of thick cutter is 12 mm, thickness of thick cutter is 3 mm, edge angle of thick cutter is 20°, width of thin cutter is 12mm, and thickness of thin cutter is 0.3 mm.

Typical curve of cutting force performed by factors on level 3 is shown in Fig 5. It shows that the cutting force increased gradually as cutters cut into banana crown and reached the maximum then decreased rapidly. The useful power consumption which means power consumed by the cutters in cutting process could be calculated based on the curve of cutting force on displacement.

### 3.2 Grade of cut surface quality

#### 3.2.1 Very bad

**Fig 6:**
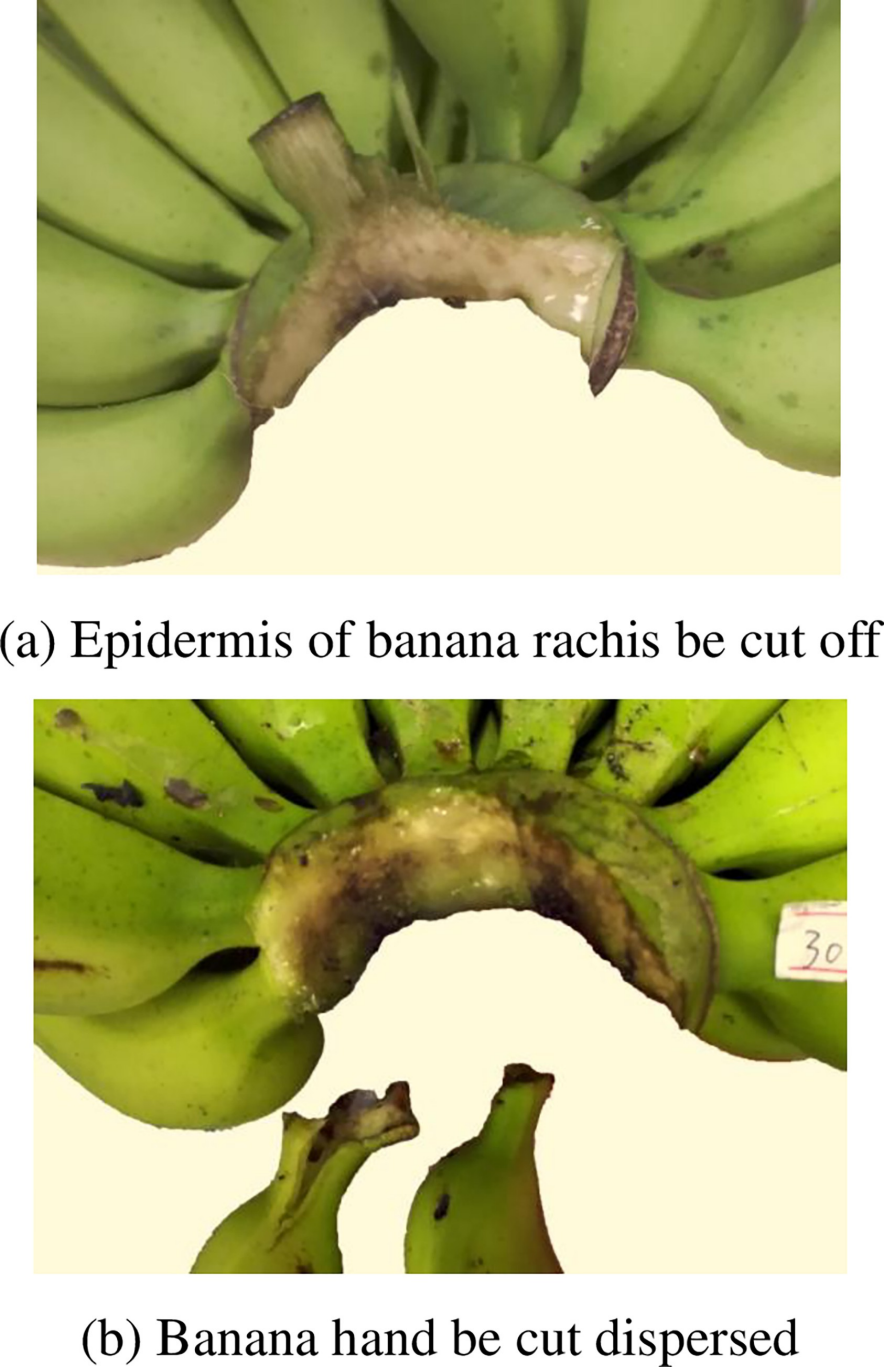
Cut surface quality of banana crown at grade 60–69. (a) Epidermis of banana rachis be cut off, (b) Banana hand be cut dispersed.

During the cutting process, if the banana peel is damaged, the banana finger separated from banana hand by the cutter, the cut surface quality grade will be very bad for banana preservation, so these cut surface of banana crown is very bad and be graded 60–69 (Fig 6).

#### 3.2.2 Bad

**Fig 7:**
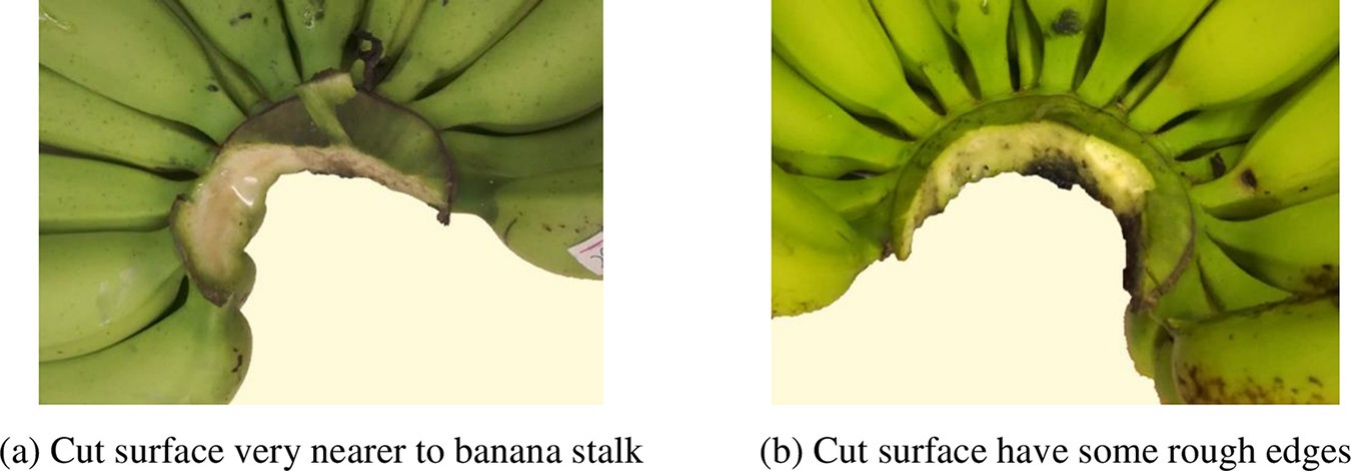
Cut surface quality of banana crown at grade 70–79. (a) Cut surface very nearer to banana stalk, (b) Cut surface have some rough edges.

If there is no skin from the rachis be cut off and banana fingers not separated from banana hand by the cutter, but the cut surface have some rough edges and very nearer to banana stalk, the cut surface quality grade is also bad for banana preservation, so these cut surface of banana crown is bad and be graded 70–79 (Fig 7).

#### 3.2.3 Good

**Fig 8:**
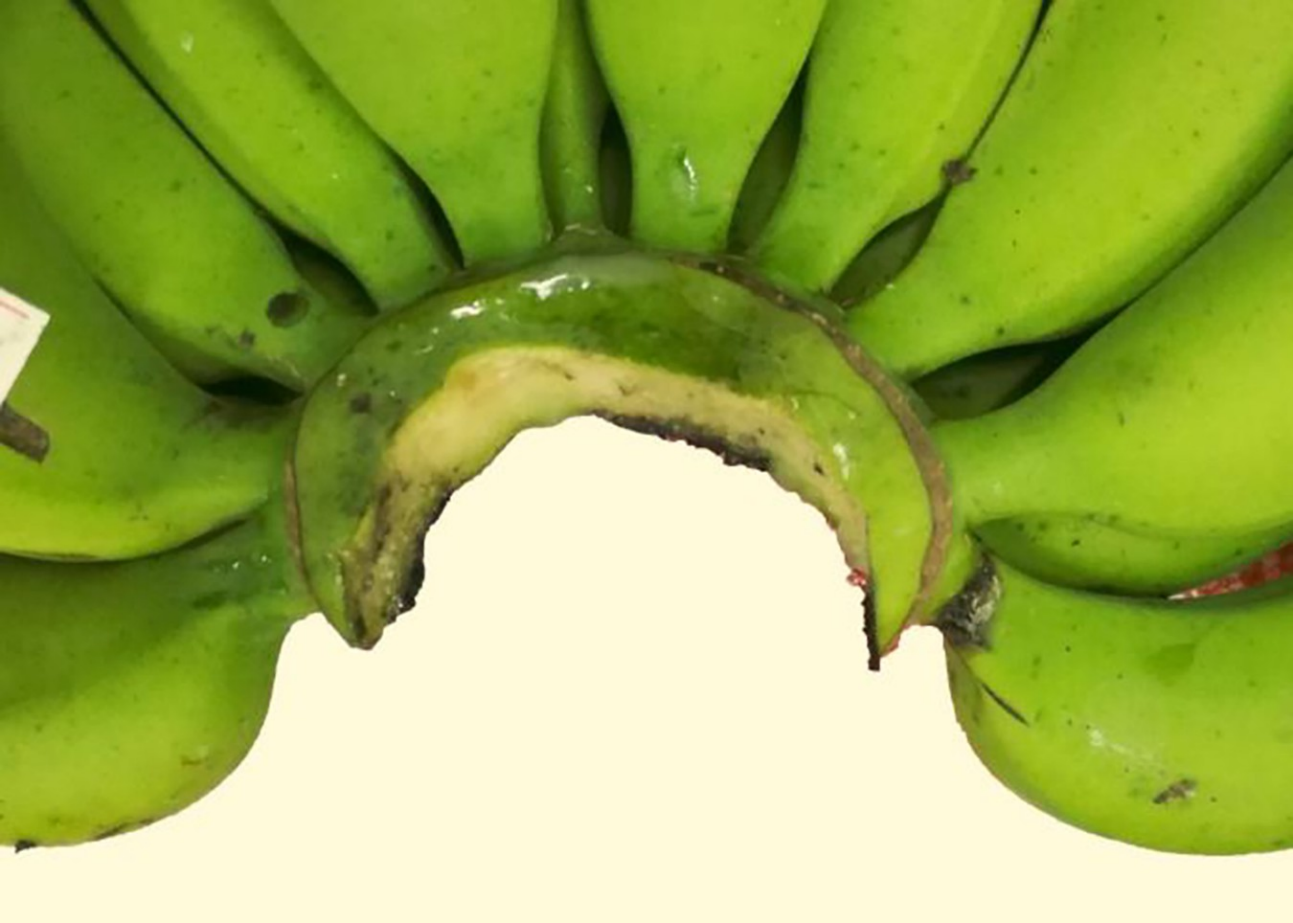
Cut surface quality of banana crown at grade 80–89.

If there is no skin from the rachis be cut off and banana fingers not separated from banana hand by the tool, and the cut surface have little rough edges and not very nearer to banana stalk, the cut surface quality grade is good for banana preservation, so these cut surface of banana crown is good and be graded 80–89 (Fig 8).

#### 3.2.4 Very good

**Fig 9:**
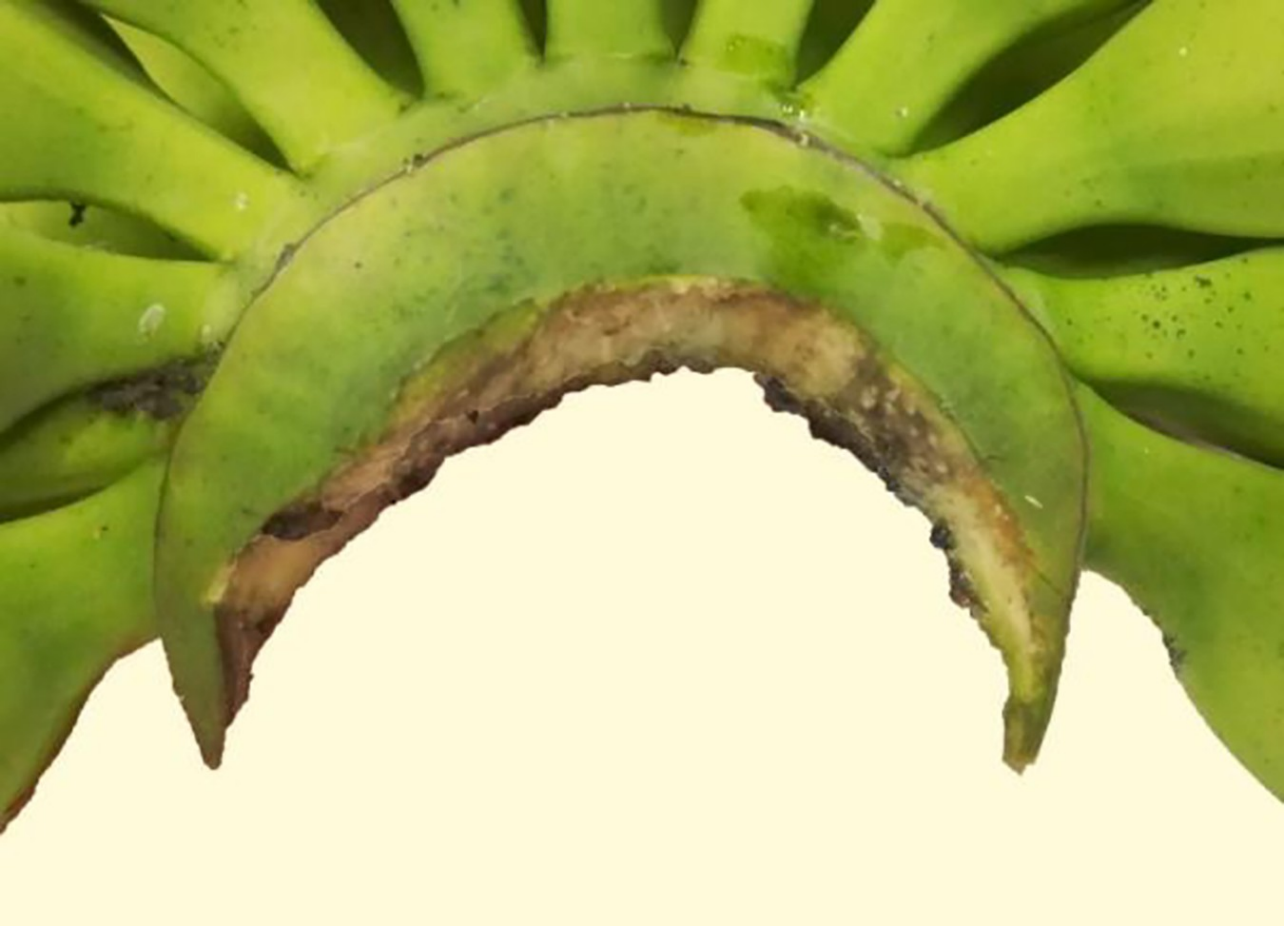
Cut surface quality of banana crown at grade 90–100.

If there is no skin from the rachis be cut off and banana fingers not separated from banana hand by the tool and the cut surface have no rough edge and not nearer to banana stalk, the cut surface is very good for banana preservation, so the quality of these cut surface of banana crown is very good and be graded 90–100 (Fig 9).

### 3.3 Indexes on number of cut sets

**Fig 10:**
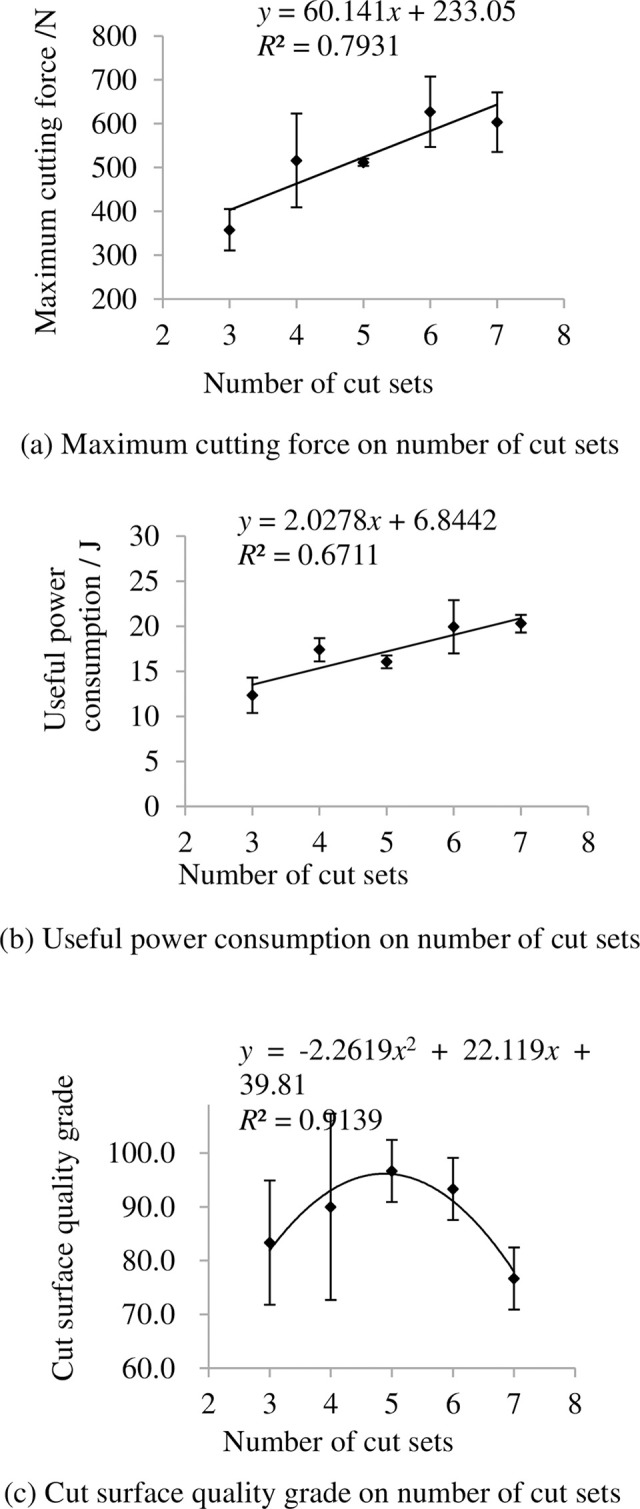
Indexes on number of cut sets. (a) Maximum cutting force on number of cut sets, (b) Useful power consumption on number of cut sets, (c) Cut surface quality grade on number of cut sets. Note: other factors were at level 3.

The indexes on number of cut sets are shown in Fig 10A–10C. It shows that the maximum cutting force and useful power consumption increase with increasing number of cut sets. The main reason is that thick cutter consumes more energy than thin cutter. The grade of cut surface quality grade first increase then decrease with increasing number of cut sets.

### 3.4 Indexes on diameter of banana rachis

**Fig 11:**
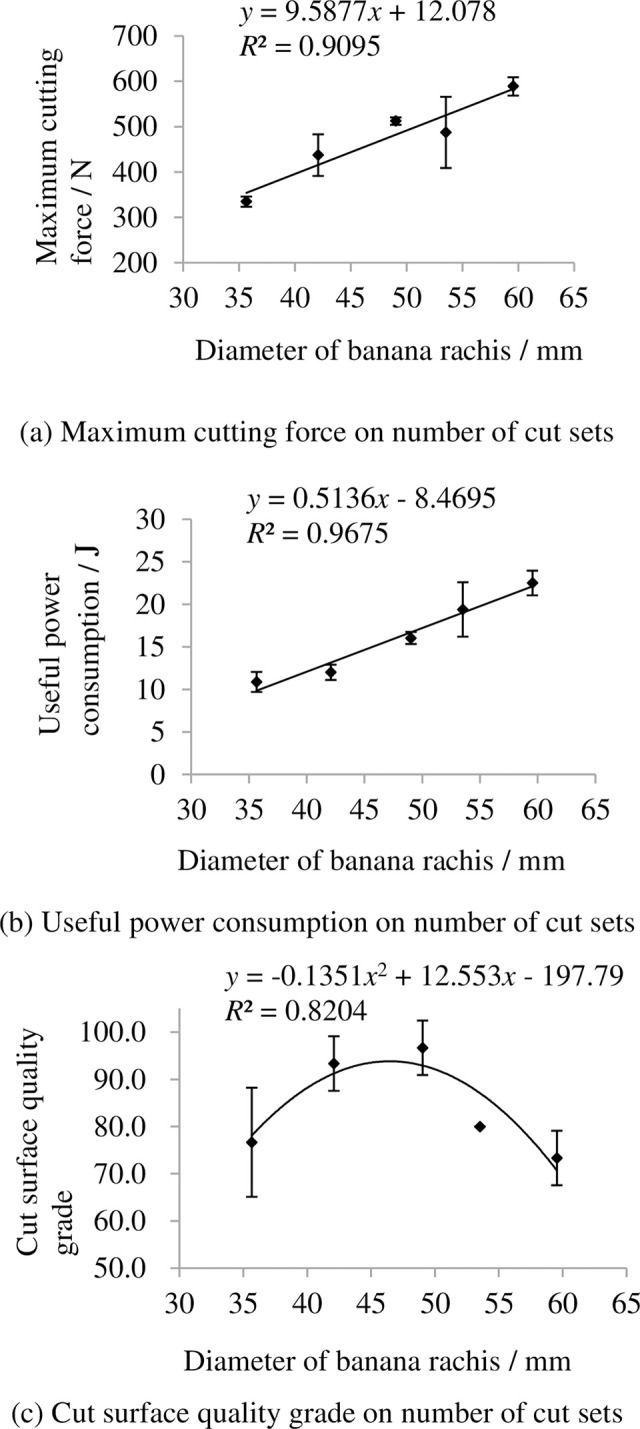
Indexes on diameter of banana rachis. (a) Maximum cutting force on number of cut sets, (b) Useful power consumption on number of cut sets, (c) Cut surface quality grade on number of cut sets. Note: other factors were at level 3.

The indexes on diameter of banana rachis are shown in Fig 11A–11C. It shows that the maximum cutting force and useful power consumption increase with increasing diameter of banana rachis. This is mainly because big rachis diameter means banana hand have more fingers and banana crown grow much stronger, so it needs more power to be cut off. The grade of cut surface quality grade first increase then decrease with increasing diameter of banana rachis.

### 3.5 Indexes on cutting speed

**Fig 12:**
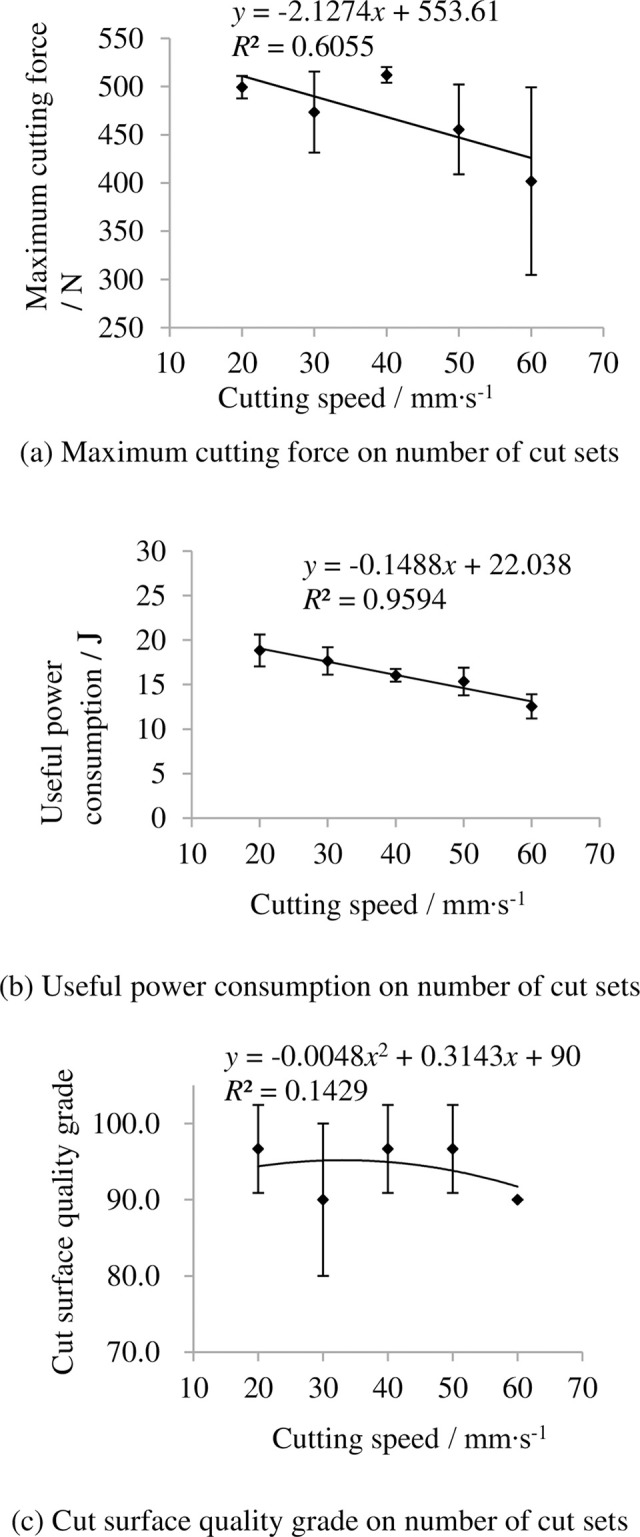
Indexes on cutting speed. (a) Maximum cutting force on number of cut sets, (b) Useful power consumption on number of cut sets, (c) Cut surface quality grade on number of cut sets. Note: other factors were at level 3.

The indexes on cutting speed are shown in Fig 12A–12C. It shows that the maximum cutting force and useful power consumption decrease with increasing cutting speed and the grade of cut surface quality grade changed not too much with increasing cutting speed between 20 and 40 mm/s.

### 3.6 Indexes on angle between thick cutter and axis of banana rachis

**Fig 13:**
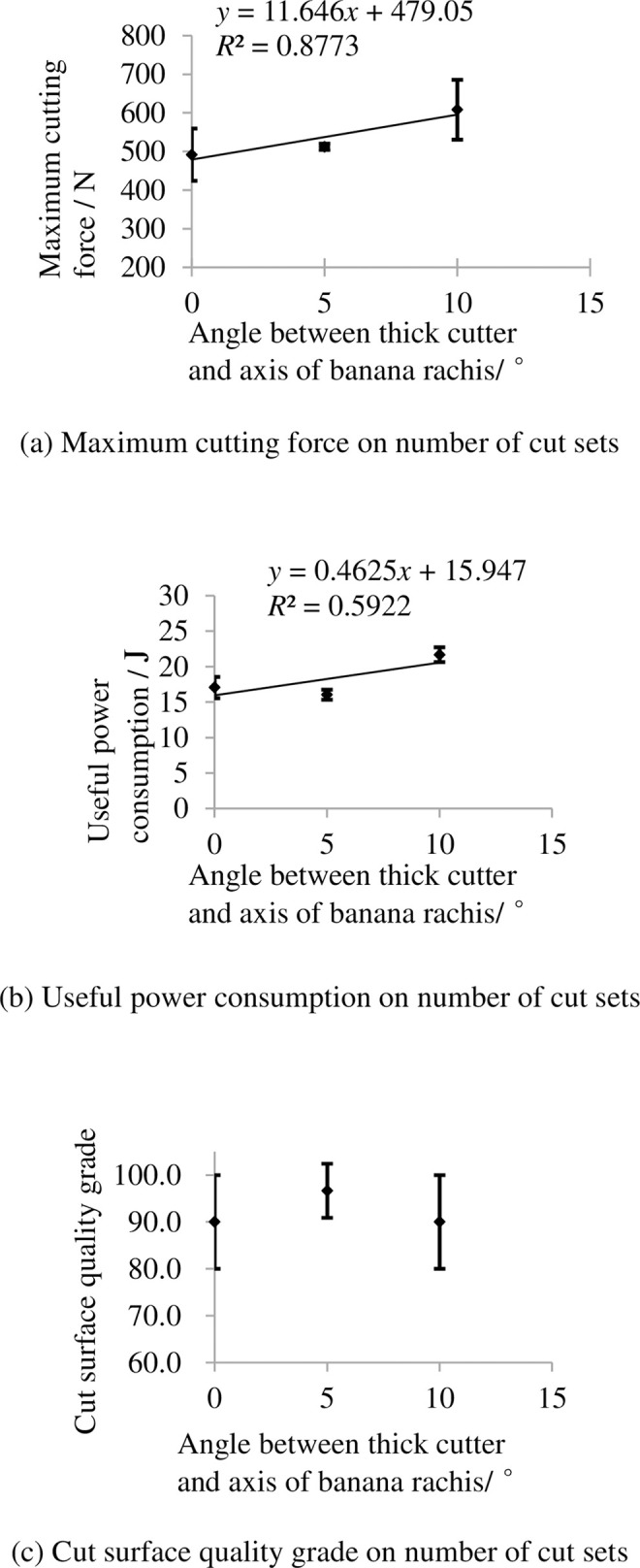
Indexes on angle between thick cutter and axis of banana rachis. (a) Maximum cutting force on number of cut sets, (b) Useful power consumption on number of cut sets, (c) Cut surface quality grade on number of cut sets. Note: other factors were at level 3.

The indexes on angle between thick cutter and axis of banana rachis are shown in Fig 13A–13C. It shows that the maximum cutting force and useful power consumption increase with increasing angle between thick cutter and axis of banana rachis. This is mainly because the angle increase means the thick cutter consume more energy in compressing the rachis in cutting treatment. The grade of cut surface quality grade first increase then decrease with increasing angle because the upper side diameter of rachis is bigger than the down side and little but not too much angle is appropriate.

### 3.7 Indexes on edge angle of thick cutter

**Fig 14:**
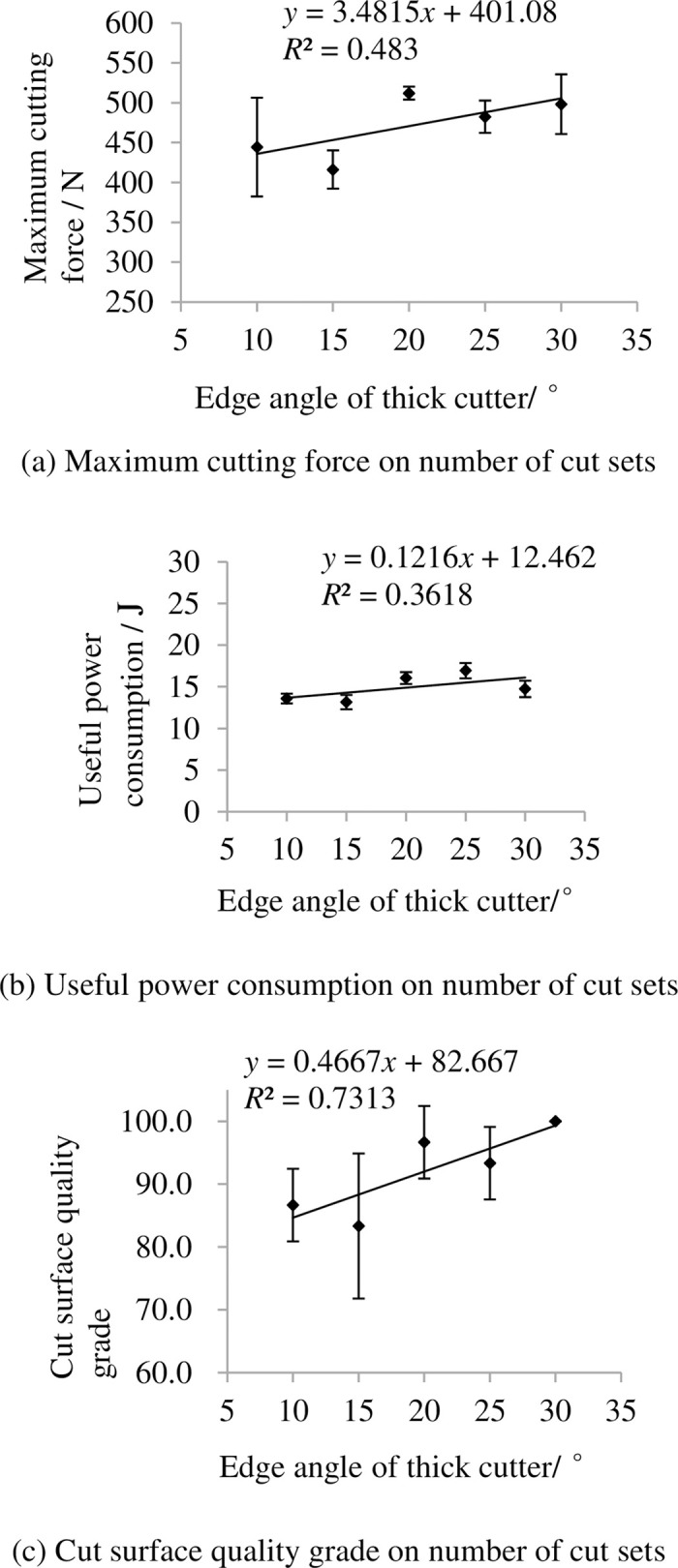
Indexes on edge angle of thick cutter. (a) Maximum cutting force on number of cut sets, (b) Useful power consumption on number of cut sets, (c) Cut surface quality grade on number of cut sets. Note: other factors were at level 3.

The indexes on edge angle of thick cutter are shown in Fig 14A–14C. It shows that the maximum cutting force and useful power consumption changed not too much with increasing edge angle of thick cutter. The grade of cut surface quality grade increase with increasing edge angle of thick cutter because when the angle is less than 20° the skin from the rachis will be cut off by the thick cutter.

### 3.8 Indexes on width of thick cutter

**Fig 15:**
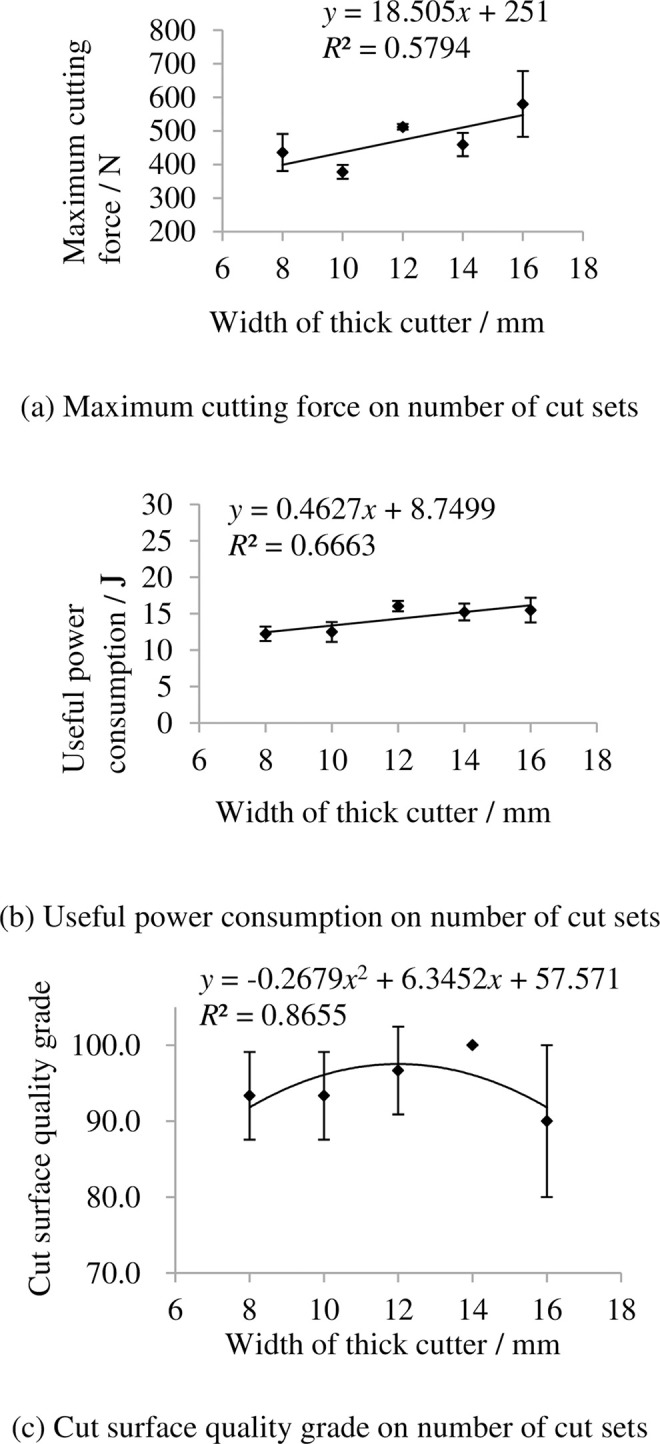
Indexes on width of thick cutter. (a) Maximum cutting force on number of cut sets, (b) Useful power consumption on number of cut sets, (c) Cut surface quality grade on number of cut sets. Note: other factors were at level 3.

The indexes on width of thick cutter are shown in Fig 15A–15C. It shows that the maximum cutting force and useful power consumption increase with increasing width of thick cutter. This is mainly because the thick cutter will consume more energy in cutting treatment when it becomes wider. The grade of cut surface quality grade first increase then decrease with increasing width of thick cutter.

### 3.9 Indexes on thickness of thick cutter

**Fig 16:**
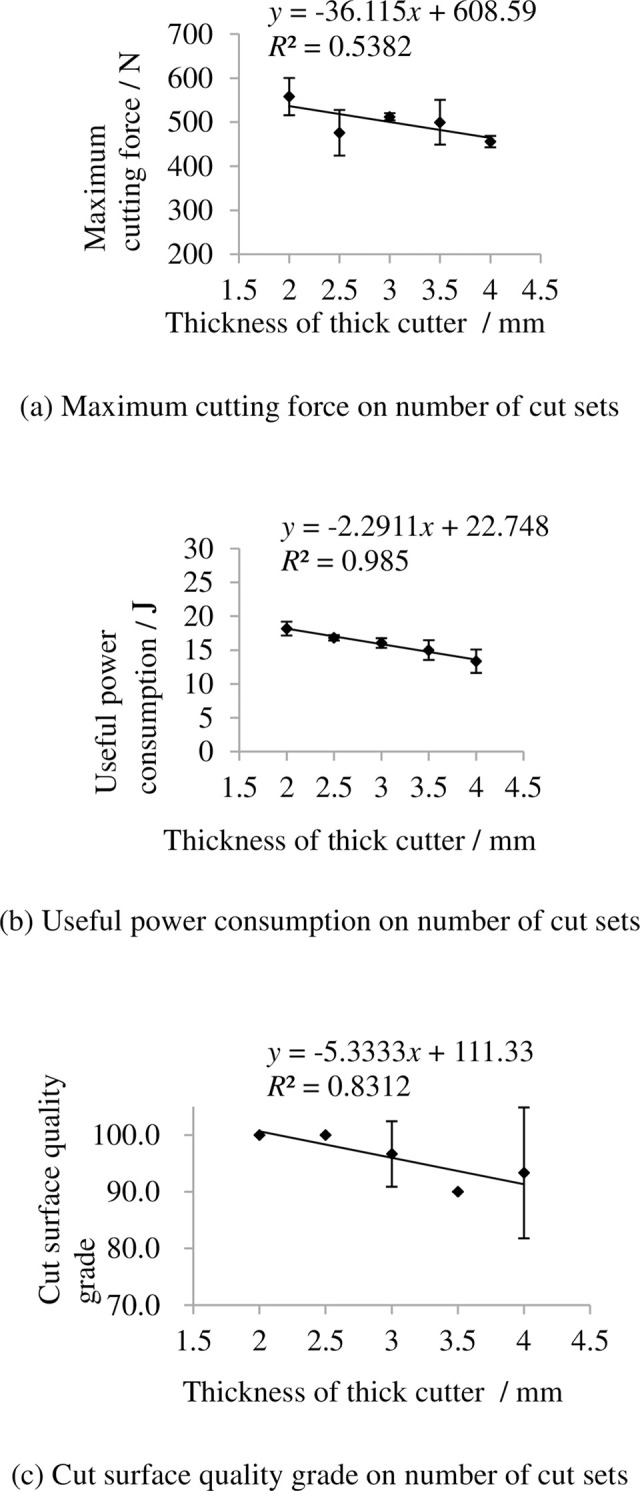
Indexes on thickness of thick cutter. (a) Maximum cutting force on number of cut sets, (b) Useful power consumption on number of cut sets, (c) Cut surface quality grade on number of cut sets. Note: other factors were at level 3.

The indexes on thickness of thick cutter are shown in Fig 16A–16C. It shows that the maximum cutting force and useful power consumption decrease with increasing thickness of thick cutter mainly as the thick cutter is tightly compress the rachis and the thickness of rachis is reduced gradually when the thickness of thick cutter changed from 2–4 mm. The grade of cut surface quality grade decrease with increasing thickness of thick cutter because thickness of thick cutter increase means the cut surface will nearer to banana stalk.

### 3.10 Indexes on width of thin cutter

**Fig 17:**
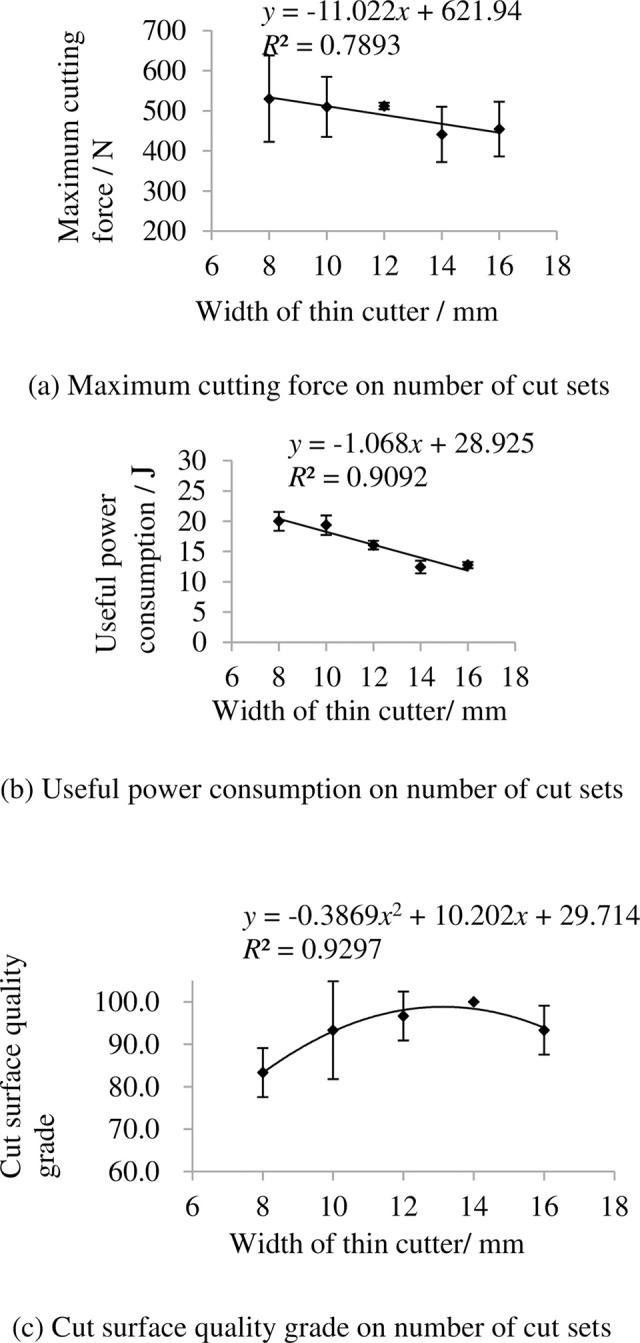
Indexes on width of thin cutter. (a) Maximum cutting force on number of cut sets, (b) Useful power consumption on number of cut sets, (c) Cut surface quality grade on number of cut sets. Note: other factors were at level 3.

The indexes on width of thin cutter are shown in Fig 17A–17C. It shows that the maximum cutting force and useful power consumption decrease with increasing width of thin cutter mainly because wider thin cutter will be more stable in cutting treatment. The grade of cut surface quality grade first increase then decrease with increasing width of thin cutter as the thin cutter is curved and wider cutter than 14 mm means resistance increases, but if the width of thin cutter is less than 12 mm it will result in elastic deformation.

### 3.11 Indexes on thickness of thin cutter

**Fig 18:**
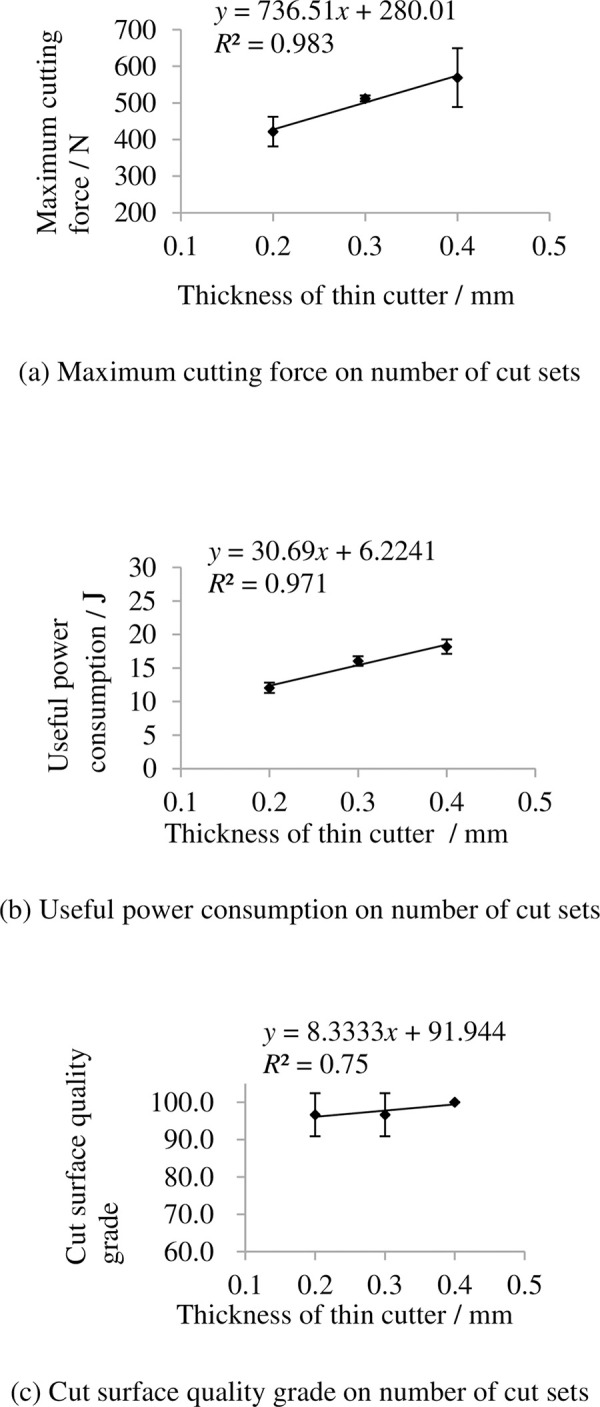
Indexes on thickness of thin cutter. (a) Maximum cutting force on number of cut sets, (b) Useful power consumption on number of cut sets, (c) Cut surface quality grade on number of cut sets. Note: other factors were at level 3.

The indexes on thickness of thin cutter are shown in Fig 18A–18C. It shows that the maximum cutting force and useful power consumption increase with increasing thickness of thin cutter. This is mainly because resistance increases as the thin cutter become thicker. The grade of cut surface quality grade changed not too much with increasing thickness of thin cutter because the thin cutter not changed too much.

## 4. Discussion

The banana mechanical crown cutting tool by combined cutters way is a critical component of banana crown cutting machine which requires less energy consumption and get high grade of cut surface quality. For banana fresh preservation, get high grade of cut surface quality is more significant than consume less energy. Results of experiments show that the optimum parameters are as follows: cutting speed is about50-60 mm/s, number of cut sets are about 4–6, angle between thick cutter and axis of banana rachis is about 5°, width of thick cutter is about 8–14 mm, thickness of thick cutter is about 2–3 mm, edge angle of thick cutter is between 20° and 30°, width of thin cutter is about 10–14 mm and thickness of thin cutter is about 0.2–0.4mm.
